# Patient Experiences of a Group Intervention Integrating Vestibular Rehabilitation, Body Awareness, and Cognitive Behavioral Therapy for Long-Lasting Dizziness: A Focus Group Study

**DOI:** 10.1093/ptj/pzaf062

**Published:** 2025-04-30

**Authors:** Liv Heide Magnussen, Kjersti Thulin Wilhelmsen, Målfrid Råheim

**Affiliations:** Department of Health and Functioning, Western Norway University of Applied Sciences, 5020 Bergen, Norway; Department of Health and Functioning, Western Norway University of Applied Sciences, 5020 Bergen, Norway; Department of Global Public Health and Primary Care, University of Bergen, 5020 Bergen, Norway

**Keywords:** Body Awareness, Cognitive Behavioral Therapy, Focus Group, Long-Lasting Dizziness, Vestibular Rehabilitation

## Abstract

**Objective:**

Long-lasting dizziness is a distressing and disabling condition frequently accompanied by psychological and physical discomfort, and if untreated, could evolve into a complex, self-perpetuating condition challenging treatment. A treatment approach addressing psychological, physical, and social ailments in connection with long-term dizziness has been developed. The objective of this study was to explore experiences and perceptions of participants with long-lasting dizziness who have engaged in a group-based intervention approach that combines principles from vestibular rehabilitation (VR), body awareness therapy (BA), and cognitive behavioral therapy (CBT) in primary care.

**Methods:**

The study is rooted in an interpretative approach. Fifteen participants, 10 women and 5 men, aged 38 to 71 years, were interviewed in 3 focus groups. Data were analyzed by systematic text condensation, a 4-step thematic cross-case strategy suitable for exploratory investigations.

**Results:**

Four main themes emerged from the analyses: (1) to share and feel understood when struggling with dizziness; (2) the exercises: body perceptions and challenging one’s own limits to control dizziness; (3) increased self-knowledge helps to process anxiety and challenge avoidance behavior; (4) changing habits is hard work, but necessary to recover from dizziness.

**Conclusions:**

This novel group-based VR-BA-CBT treatment for individuals with long-lasting dizziness offered valuable peer support, shared learning, and learning in action providing new understanding. The VR-BA-CBT treatment includes a comprehensive and holistic approach addressing physical, psychological, and social challenges.

**Impact:**

Through knowledge about dizziness triggers, participants learn new strategies to confront previously avoided activities. The approach holds promise to be implemented in primary care physical therapy settings.

## INTRODUCTION

Vestibular-related dizziness lasting longer beyond 3 months affects 29% to 50% of individuals with vestibular disorders.^1–3^ This prolonged dizziness often leads to psychological distress, anxiety,^4^ avoidance behaviors,^5^ and musculoskeletal pain.^6–8^ The condition may become self-perpetuating,^9^^,^^10^ making it challenging to treat.

Vestibular rehabilitation (VR) is an exercise-based approach, often combined with educational components, designed to help individuals overcome dizziness and balance disturbances.^11^ According to clinical guidelines, VR is the preferred treatment for unilateral hypofunction.^12^^,^^13^ Recent studies also show benefits of VR for patients with persistent postural-perceptual dizziness (PPPD)^14–16^ and for chronic dizziness.^16^ Vestibular rehabilitation exercises promote vestibular compensation, a central learning process,^17^ by addressing gaze stabilization, habituation, and postural control.^12^^,^^13^ Gaze stabilization involves adaptation inducing long-term changes in the neural response to head movements while substitution encourages the use of intact sensory inputs (visual, somatosensory) over dysfunctional ones (vestibular). Habituation reduces responsiveness to motion stimuli, alleviating dizziness symptoms.

Anxiety is common in individuals with long-lasting dizziness, perpetuating symptoms and leading to fear, frustration, and hopelessness.^1^^,^^18^^,^^19^ Combining VR with cognitive behavioral therapy (CBT) was proposed to address these psychological factors.^20^ Cognitive behavioral therapy posits that cognitive factors sustain psychological distress, encouraging patients to identify and challenge maladaptive thoughts to alter negative behaviors.^21^ Useful techniques in prolonged dizziness include psychoeducation exploring links between thoughts, behaviors, exposure to feared stimuli, attentional refocusing, coping strategies, and self-observation.^20^^,^^22^^,^^23^ Previous studies combining VR-CBT have demonstrated promising results in reducing dizziness-related symptoms, but have methodological shortcomings and small sample sizes.^24^^,^^25^ A recent randomized feasibility study found that integrated CBT-based VR showed better results than standard VR for patients with PPPD.^26^ Follow-up observations after multimodal treatments combining VR, CBT, and medication indicate symptom reduction in chronic dizziness.^16^^,^^27^ However, well-powered randomized controlled studies evaluating the efficacy of such programs are lacking.^16^ In Europe, access to and knowledge of evidence-based treatment is scant.^28^ To increase capacity and educate physical therapists in primary care is necessary.

A group-based intervention combining VR, body awareness (BA), and CBT was developed to address Long-lasting Dizziness In Primary Care (LODIP).^29^^,^^30^ Body awareness, rooted in phenomenology, involves a pre-reflective dimension in movement and sensations. When functioning well, the body is present (eu-appears) in experience without conscious awareness of movement or sensation.^31^^,^^32^ Experience is embodied, shaping our engagement with the environment and others.^31^^,^^33^ Body awareness therapies aim to alter habitual patterns like muscle tension to improve physical ailments, stress, and overall well-being. Gaze stability and habituation exercises, integrated with BA into daily movements, become a source of direct learning.^34^ Participants reflect on bodily sensations during exercises and share insights through guided self-reflection. The CBT component aims to disrupt the anxiety cycle triggered by the “fight or flight” response leading to catastrophic misinterpretations and safety-seeking behaviors.^22^ Cognitive behavioral therapy challenges avoidance strategies to build confidence and resilience. The group format aligns with Social Cognitive Learning Theory emphasizing learning from observing and interacting with peers.^34^ Cognitive factors like beliefs and expectations influence behavioral change through direct experiences, peer support, and symptom changes during activities leading to self-efficacy.^35^ Feedback from the feasibility study^29^ indicated the intervention’s feasibility and improvement in function.

Research on participants’ lived experiences with multimodal interventions for long-lasting dizziness is limited. Understanding these experiences is crucial for improving and tailoring treatment. This study aims to explore the experiences and perceptions of participants after undergoing this innovative group-based intervention in primary care settings.

## METHODOLOGY, METHODS, AND PARTICIPANTS

This study is informed by philosophical hermeneutics, focusing on the interpretation of texts, conversations, acts, events, and cultural artifacts.^36^ In Gadamer’s terms, understanding is influenced by pre-understandings embedded in tradition and language, which are social and historical in nature.^37^ This inherent pre-understanding shapes both human interpretation and research. Interpreters (researchers) bring their pre-understandings, that is, knowledge, expectations, and experiences with the subject matter, situating their understanding in concrete contexts (here, a new treatment program for patients with long-lasting dizziness in a Norwegian research project). Gadamer^37^ distinguishes between positive and negative pre-understandings (prejudice). The first facilitates new understanding, while the last risks misunderstanding. Therefore, reflexive practice within the research team is essential. Gadamer^37^ emphasizes actively putting own prejudices at risk to remain open to the other (the participants), to the unexpected (findings). In philosophical hermeneutics, understanding is a dynamic process involving a continuous interplay between parts and whole, text and context, and pre-understanding and new understanding, termed the hermeneutical circle or spiral.^36^ This approach is applied when analyzing interview tapes and transcripts, revisiting and reconsidering patterns and themes, and contextualizing and theorizing the findings in the discussion.

Focus group interviews were conducted with participants from the LODIP study, an 8-week group-based intervention incorporating VR, BA, and CBT.^30^ LODIP included 107 participants (70% women) recruited from primary care, age between 18 and 70 years, with acute-onset and long-lasting dizziness (>3 months), worsened by head movements. Participants were informed about the qualitative study through the LODIP consent letter. Focus groups were chosen to capture group dynamics and interconnections efficiently.^38^ Eligible participants completed at least 6 of 8 group treatment sessions; 40 participants met this criterion. To ensure diversity, both genders, varying ages, and backgrounds were selected.^38^ Fifteen participants (10 women) aged 38 to 71 years representing all 8 treatment groups agreed to participate. The interviews, lasting for 100 to 115 min, took place at Western Norway University of Applied Sciences from September 2019 to January 2020, 3 to 4 months after their final treatment session.

### Data Collection

Data generation was conducted collaboratively by the lead (L.H.M.) and the co-researcher (M.R.), serving as facilitators for the focus group interviews, L.H.M. as moderator and M.R. as co-moderator. To ensure comprehensive documentation, M.R. took field notes during the interviews, which contributed to the analysis. The focus group interviews took place in a meeting room. A semi-structured interview guide, consisting of open-ended questions, was utilized. The questions covered experiences with the educational component, group interactions, adherence to home-based exercises, reflections on the exercises, and perceptions of the group format. Before starting the interviews, the moderators informed that all experiences, negative and positive, were important. The interviews were audio-recorded, and the verbatim transcriptions served as the basis for analysis. To maintain confidentiality and anonymity, pseudonyms were assigned to the participants in the transcribed records.

### Data Analysis

We chose systematic text condensation, a thematic cross-case strategy suitable for exploratory analysis of qualitative data. Systematic text condensation systematizes a dynamic process alternating between part and whole in the interview text.^39^ The analytic process involved 4 main steps. First, preliminary themes were identified through repeated readings of the text. Second, text segments with shared meanings regarding the group intervention experiences were identified as meaning units and organized into code groups. Third, code groups and subgroups were refined, and condensed insights were extracted from each group. This step aimed to capture a condensed essence of participants’ experiences. Finally, the condensed insights were re-conceptualized into synthesized descriptions capturing the range of participants’ experiences. To ensure methodological rigor, a collaborative approach was taken, the authors engaging in discussions to reach consensus and a shared understanding. Identification of overarching themes relevant to the study’s aim was done collaboratively. Recurrent comparisons were made between emergent themes and the original dataset, with additional insights from the field notes enriching the analysis.

#### Group-Based Intervention Combining VR, BA, and CBT

The intervention (Table), led by 2 trained physical therapists, was carried out in primary care in eight 2-hour sessions, 5 to 8 participants in each group.^30^ The first 3 sessions had a widened focus on psychoeducation, while the subsequent 5 sessions placed more emphasis on physical exercise.

**Table:** Description of the Treatment Sessions in the Group-Based VR-BA-CBT Program*^a^*

**Sessions**	**Focus**	**Examples of Tasks/Exercises**
1	Dizziness and additional/secondary complaints	Discussion on dizziness and additional complaints. Introducing the vicious circle that can arise between somatic symptoms and the catastrophic misinterpretation of these. Exercises: Body awareness in sitting and standing. Habituation exercises.
2	The “vicious circle”	How somatic symptoms related to dizziness and anxiety can be appraised appropriately by mapping the relevant symptoms, thoughts, and potential avoidance behavior individually. Introducing the “fight or flight” response and how this may be relevant for chronic dizziness. Exercises: Body awareness in standing and walking. Habituation and gaze stability through games (planned and unplanned head turns with gaze fixation). Relaxation.
3	The “fight or flight response”	Discussion regarding experiences related to the fight or flight response. Is the alarm needed? How can these symptoms be appraised in relation to persistent dizziness? Exercises: Habituation, gaze stability, and body awareness (standing balance, walking with directional changes). Reflection during and after exercises. What happened? What was your response? (in every session from here). Relaxation.
4	The fight or flight response and management	Discussion: How did you respond to the fight or flight response in everyday life following the last session? Individual goal setting. Exercises: Habituation, gaze stabilization, walking, ball games changing place, turns, and rotations. Relaxation.
5	Relaxation	Discussion/reflection: Exercises, dosage, and “relaxation.” It is normal to be dizzy and tired after exercises. Exercises: Gaze stability and habituation games (eg, practice with ball hitting various targets, bowling). Working alone and in pairs. Relaxation.
6	Movement-induced dizziness	Any changes in relation to the “vicious circle” described in first session? Group and individual reflection. Exercises: Habituation games in larger groups and in pairs. Walking with head rotations, velocity changes, externally induced stop/start. Relaxation.
7	What next? Preparation for the future	Discussion before, individual reflection during, and group reflection after exercises: “How do I cope/deal/manage the dizziness? What thoughts are formed when I get dizzy?” Exercises: Combination of balance and habituation, activities and games in groups and in pairs (eg, obstacle course, standing back-to-back, passing ball at different heights). Relaxation.
8	Reflection and conclusion	Discussion: “What have I learnt? What do I take with me? What do I do when/if dizziness returns?” Exercises: Balance and body awareness in standing and walking, changing directions, different velocities, stop/start. Gaze stability and habituation. Ball activities alone, in pairs, and in larger group. Relaxation.

^a^ Reproduction of the table with permission from BMC Trials, Springer Nature. Table is published in the protocol study.^30^ Abbreviations: VR-BA-CBT = vestibular rehabilitation, body awareness, and cognitive behavioral therapy.

##### Ethics

Ethical approval was granted by the Regional Committee for Medical and Health Research Ethics (2014-00921). COREQ guidelines were used in the conduction of the qualitative study.

### Role of the Funding Source

The funders played no role in the design, conduct, or reporting of this study.

## RESULTS

Four main themes emerged: (1) To share and feel understood when struggling with dizziness; (2) The exercises: body perceptions and challenging one’s own limits to control dizziness; (3) Increased self-knowledge helps to process anxiety and challenge avoidance behavior; (4) Changing habits is hard work, but necessary to recover from dizziness.

### Theme 1: To Share and Feel Understood When Struggling With Dizziness

The participants found it crucial to interact with peers who shared similar experiences of enduring persistent dizziness. They expressed a sense of navigating a complex system where neither health care practitioners nor family and friends fully comprehended the extent of their challenges. Exchanging experiences provided them with a sense of relief and a feeling of being understood. Some even reported improved well-being after the first session.

The participants often perceived themselves as “different” due to their unsteady movements and frequently contemplated the opinions of others. Within the groups, participants found solace in sharing experiences that others might find strange, such as “the disorienting tiles in a shopping mall that triggered intense dizziness” (Ann). Acknowledging these shared experiences fostered a sense of camaraderie. They openly discussed their challenges without judgment, which alleviated feelings of isolation and being different. A woman in her late sixties expressed:

Just like me! Sharing experiences made me realize I wasn’t alone in feeling stressed and overwhelmed. It solved my misconception that these feelings were exclusive to females. Connecting and conversing with others had a significant positive impact on me. (Esme)

Several participants experienced a pervasive sense of hopelessness regarding their dizziness, but the intervention instilled optimism for recovery. Witnessing the progress of participants facing greater limitations fueled their own aspiration and hope. They highlighted the motivational influence of collective engagement and group activities which provided mutual support, encouragement, and empowerment. As a result, they developed a belief in the possibility of escaping their trapped situation. One participant eloquently expressed this sentiment:

I gained hope that this phase would eventually pass, and I’d recover. Witnessing others in more challenging situations than mine recover, was comforting and gave me sense of security. (John)

During the sessions, the participants observed fellow members successfully navigating exercises that induced dizziness, which resulted in improvements. The group celebrated these successes together, inspiring each other and fostering a sense of belief in their own abilities*.* One participant who was initially hesitant to join a trip due to concerns about travel-related discomfort decided to go after receiving encouragement. The experience turned out positive; “Pushing myself despite fear of dizziness is indeed practicing.” (Henry).

### Theme 2: The Exercises: Body Perceptions and Challenging One’s Own Limits to Control Dizziness

Participants found the tailored exercises to be highly beneficial despite experiencing temporary exacerbation of dizziness. They reported quick symptom relief after performing the exercises. The exercises also increased their awareness of bodily sensations enabling them to differentiate between feeling “off balance” and “dizzy” in everyday situations. Several participants mentioned experiencing sensations such as neck pain, muscle tension, stiffness, and a feeling of moving “en block.” They realized that pain, tension, and dizziness could reinforce each other. Relaxation exercises, including 1 that focused on tongue position to release jaw tension, helped them become more aware of these interactions.

The exercises that induced dizziness provided participants with insights into their personal triggers and prompted them to reflect on activities they had avoided due to fear. Through systematic testing of triggers and repeated confrontation via activities, participants experienced dizziness improving. The process also contributed to increase confidence and a sense of control. “I feel like I’m now in control of my dizziness, it’s not the dizziness that controls me” (Mary). Individualized goals and mutual commitment in the group facilitated and motivated to self-training between sessions. The group members’ enthusiasm was an open invitation to share their achievements upon returning to the sessions.

I trigger dizziness by looking up and down on marks placed on the wall. Despite feeling dizzy and nauseous, I persisted, keeping track of repetitions. I shared this with the group. (Thea)

Participants seamlessly integrated exercises into their daily lives, like head turns while hanging up laundry, a task which typically triggers dizziness. By persevering despite dizziness, they experienced a sense of mastery as it subsided. Incorporating provoking movements into practical tasks helped them overcome challenges and prevent future attacks. Repeating these movements and exercises heightened their awareness of their own bodily reactions, allowing them to make necessary changes for better control. They began to see dizziness as a useful tool for reducing its effects. Using dizziness as a guide for their actions and movements empowered them.

I’ve become highly aware. While walking, I choose landmarks, practice turns. I ensure no one’s behind as it feels odd. Climbing stairs is deliberate practicing, and if I feel dizzy, I remind myself that I’m “in it”, this is practice. I’m attentive to minor details. (Henry)

### Theme 3: Increased Self-Knowledge Helps to Process Anxiety and Challenge Avoidance Behavior

Understanding that dizziness was not harmful or dangerous was a pivotal realization for participants and improved their ability to live with the condition. Group discussions played a crucial role in encouraging reflections on daily life, including identifying dizziness triggers, coping methods, and reactions Many identified daily activities they avoided, finding value in addressing these situations by practicing alternative strategies. The group leaders emphasized the connection between movement strategies, avoidance, and dizziness. They repeatedly and skillfully guided participants in confronting and practicing new strategies and facilitated peer support. Participants recognized that the combination of knowledge, support, and exposure was the key to success in managing dizziness.

Knowledge matters. Despite reading and online searches, gaining insights from those possessing factual knowledge, is vital for progress. (John)

Recognizing stress as a trigger of dizziness, empowered participants take control by pausing and practicing relaxation techniques when they felt its onset. They realized that altering situations and ingrained habits was crucial for managing dizziness. Daily situations that previously were avoided, were identified and addressed.

Understanding that “getting dizzy” is not dangerous, is crucial. Embracing exposure to dizziness is valuable. It’s a mental concept too, anticipating dizziness can be misleading. I used to wait for it but realized that it was just in my mind. (Hannah)

Learning about the “vicious circle” was beneficial for most, although a few did not relate to the concept. Still, several admitted to feeling anxious about dizziness, making them apprehensive to specific movements and situations. “I avoided turning my head upon waking out of fear of dizziness” (Hannah). Recognizing the necessity to confront and dismantle the avoidance patterns and discover more helpful strategies became clear. One participant shared this experience:

Positioning myself in the meeting room provided a safe space so I had a retreat, which highlighted the counterproductive nature of these strategies. I questioned their validity and explored alternative approaches, practicing outside the group sessions. (John)

Participants reflected on whether the fear of dizziness outweighed the actual experience of dizziness. They actively addressed their daily anxiety and avoidance, resulting in gradual improvement. The body’s habitual alertness and stress responses in triggering situations provided valuable information.

Understanding the body’s signals and distinguishing between reactions that worsen dizziness and those that are natural, alleviated concerns. (Mary)

Not everyone found the interplay between anxiety, stressors, bodily reactions, and dizziness relevant for their experience. Some felt that there was too much focus on psychological issues and found exercises the most beneficial component in the program. However, even those claiming “no anxiety” acknowledged that “I learned a lot from the dizziness circle and conversations” (Jane).

### Theme 4: Changing Habits Is Hard Work, but Necessary to Recover From Dizziness

Participants acknowledged the efficacy of newfound strategies for managing dizziness, but they emphasized that there was no quick fix. Progression made them forget, only to be reminded when dizziness resurfaced. This served as a reminder that establishing and upholding new habits require continuous efforts. However, as their confidence in new strategies rose, a shift was indicated, moving from vigilance to a more trustful awareness.

One teacher used to listen to music while walking across open spaces, or she walked close to walls. Her new strategy involved walking across open areas without music. Others altered their routines, such as taking alternative routes to work, positioning themselves in the center of the staff room, visiting stores they previously had avoided, or tolerating noise in a classroom. These systematic efforts expanded their comfort zones but demanded time and dedication.

Enduring sound and light [and such] disturbances after having been dizzy for a long period, was challenging. Dimming classroom lights and instructing pupils brought relief. Yet, a shift in approach emerged, I began accepting noise, permitting breaks, and enduring the cacophony. (Julia)

Participants found that when dizziness suddenly occurred, they were able to handle it effectively. The fear they once experienced was replaced by a sense of security and control. For some, conquering dizziness felt like regaining their life, allowing them to re-engage in activities like cycling and skiing without worry. They were able to walk in rough terrain, interact with others, and go shopping without feeling overwhelmed. While dizziness remained, participants felt safer and coped better, knowing what to do in such situations. They noted that consistent practice was necessary to maintain this progress. As a result, their confidence in managing dizziness increased.

## DISCUSSION

This study delves into patients’ experiences of a group-oriented CBT-informed VR-BA program. Participants expressed that engaging in the intervention instilled hope for improvement, and some reported a profound enhancement in their daily functioning. While acknowledging that complete recovery was unattainable, many emphasized the empowerment they gained from understanding dizziness triggers and learning effective countermeasures. Participants noted that the program’s exercises helped them develop self-awareness of their personal triggers and thresholds, enabling them to systematically explore their experiences and effectively manage new episodes of dizziness. Group discussions further enriched their understanding of dizziness, dispelling concerns about its physical repercussions. The integration of knowledge, support, and exposure emerged as crucial factors in improving quality of life.

### Methodological Considerations

Methods to ensure credibility in the present study included triangulation, member checking, prolonged engagement, researcher reflexivity, maintaining an audit trail, and negative case analysis.^40^ Data were generated from focus group interviews and field notes, allowing for data triangulation. Member checking was performed by providing the participants with a summary of the findings throughout the interviews to verify and nuance the interpretations. This also supported confirmability of the results. A priority was also establishing an atmosphere of trust and tranquility, avoiding imposing preconceptions. Open-ended questions and attentiveness to participants’ narratives facilitated candid sharing of experiences, resulting in rich discussions, which concerns the credibility and relevance of the data. Peer debriefing sessions among the interviewers (M.R., L.H.M.) helped to scrutinize the research process, research questions, and findings, further enhancing credibility. Preliminary thematic analysis (first step) was performed by 2 researchers separately (L.H.M., M.R., researcher triangulation), and further analysis was based on discussions leading to consensus on core themes. One of the researchers (K.W.) has profound experience in vestibular research, the others (M.R., L.H.M.) have extensive experience with qualitative research, and 1 (L.H.M.) with treating patients with long-term complex conditions, ensuring varied, relevant knowledge in the team and prolonged engagement. L.H.M. and K.W. are integral to the primary research project (LODIP study), but neither participated in the actual intervention, while M.R. was not involved in the LODIP study at all, which helped to establish analytic distance. All research decisions and core processes were discussed in the team and described to ensure transparency (audit trail). Reflexive journaling was conducted to acknowledge the researchers’ potential biases. This process supported researcher reflexivity and dependability of the results. Searching actively for cases that did not fit our initial expectations (negative case analysis) was conducted. A couple of participants expressed that they were not better from the dizziness itself, but they had nevertheless learned strategies that helped them in situations where dizziness occurred. We also used relevant theories, Bandura’s social cognitive theory and phenomenologically based theory on embodiment and body awareness, to deepen the interpretation of core findings and thus enhancing analytic validity.

Systematic text condensation was utilized for data analysis,^39^ aligning with the interpretive paradigm and offering a transparent approach. Focus group interviews capture group dynamics and interconnections among participants. Individual interviews could have provided more comprehensive insights into personal experiences, but our emphasis was on shared experiences**.** The dynamics of group interactions may influence individual responses and potentially obscure unique experiences. To mitigate this, participants were encouraged to share dissenting points of view.

To ensure diverse perspectives, 15 participants from both genders, various ages, and representing all 8 treatments groups were included in the 3 groups. Data saturation refers to the point in data collection when issues begin to be repeated and further data generation becomes redundant.^41^ We analyzed the interviews consecutively, and after the third focus group, no new thematic codes were identified. This is in line with studies showing that 80% to 90% of thematic codes are identified after 3 focus groups.^42^^,^^43^ Furthermore, sample size is determined based on information power, taking factors such as study aim, sample specificity, theory use, quality of dialogue, and analysis strategy into account.^44^ Given the focused research question and shared experiences stemming from the same treatment program, and sample specificity, the information power was considered satisfactory. The quality and relevance of the data generated in the interviews are discussed above.

### Discussion of Results

Group treatment played a crucial role in instilling hope for recovery from dizziness. Some participants experienced full recovery and a sense of reclaiming their lives, while others still struggled with dizziness. However, they found the treatment useful as they had learned strategies to cope when dizziness occurred. Persistent postural-perceptual dizziness affects spatial orientation, postural instability, and heightened motion sensitivity which impacts everyday activities,^45^ comparable to that experienced by our patients. The fear of triggering symptoms may lead to dysfunctional behaviors, gait disorders, and neck stiffness.^20^ Avoidance of activities due to fear further hampers social and professional life, as well as recovery.^45^

The intervention aimed to break the “vicious circle” of dizziness and avoidance behaviors, promoting a valuable sense of control. Beliefs about dizziness, like fear of falling and social embarrassment, can precipitate avoidance of activities perceived as threatening.^46^ Participants recognized the interplay between somatic anxiety symptoms, the misinterpretation of these symptoms, and the subsequent safety-seeking behaviors. Through systematic exposure to dizziness-inducing exercises and peer support, they learned to engage in functional strategies and gradually reduce fear. This process enabled them to overcome their self-imposed limitations. These findings align with previous research demonstrating the effectiveness of CBT in reducing dizziness-related impairment.^22^^,^^24^^,^^47^ The group-oriented, CBT-informed VR-BA program provided a supportive and empowering environment to learn strategies to improving quality of life.

The importance of the group format to enhance motivation for exercises was highlighted. Observing fellow participants’ progress had a positive impact and reflects Bandura’s concept of observational learning.^35^ Commitment to home tasks emerged from encouraging therapists and supportive peers. A sense of shared responsibility among group members further motivated the adoption of new strategies. Therapeutic relationship, knowledge, peer support, and controlled exposure were pivotal for success, factors that are acknowledged in vestibular rehabilitation.^48^

Incorporation of BA exercises aimed to increase participants’ body sensitivity and awareness in various tasks such as weight distribution, postural alignment, and breathing patterns.^8^ The participants found it useful to integrate these exercises in everyday life and registered that the exercises increased their awareness of bodily sensations, influencing thoughts, emotions, and actions. The exercises were central in enabling the participants to differentiate between bodily sensations. Augmented body awareness is shown to improve coping, function, and change in movement and breathing patterns.^49^^,^^50^ This approach emphasizes bodily experiences beyond cognitive aspects. Through dialogue and testing in safe environments, the participants gained an understanding that sensing dizziness was not dangerous.

Cognitive behavioral therapy–informed VR-BA intervention for long-lasting dizziness involves an active learning process.^17^ According to Bandura, self-efficacy^34^^,^^35^ pertains to altering maladaptive behavior linked to dizziness in our context. Self-efficacy is shaped by direct and indirect experiences, verbal persuasion, and physiological affective state, and influenced by positive or negative outcomes. Behavior is dynamic and influenced by consequences and reinforcement conditions,^34^ clearly demonstrated in this study. The VR-BA approach in CBT emphasizes understanding the interplay among thoughts, emotions, and bodily responses for behavioral change.^51^ The combined education and physical exercises in a supportive group environment boosted the participant’s self-efficacy beliefs; they could handle dizziness, which ultimately resulted in improvements. Group formats are recognized and valued from a therapeutic and cost-effective perspective.^26^^,^^29^^,^^52^^,^^53^

Indeed, it is important to acknowledge that the program’s approach went beyond a purely cognitive focus and emphasized the elevation of body awareness to facilitate transformative shifts in coping strategies. From a phenomenological perspective, habits are deeply embodied, manifesting as ingrained patterns of movement, reaction, and action in various situations.^31^ Participants recognized their habitual inclination to remain vigilant to avoid dizziness in everyday scenarios, and their tendency to avoid certain movements, tasks, and environments in response. Through the group sessions, participants deliberately induced dizziness through exercises. They were present in their own bodily experiences in a supportive environment, which allowed them to personally discover their ability to endure dizziness without retreat, eventually experiencing its decline. At home, they actively challenged established habits to avoid dizziness, gradually cultivating new responses and actions to effectively coexist or achieve full recovery. This transformative process involved the establishment of new bodily based habits, which the participants described as “hard work.” Merleau-Ponty^31^ states that the body has understood and acquired a habit when it allows itself to be influenced by and assimilate a new meaningful core. Incorporating new habits in terms of movement, reaction, and action requires time and effort, as previous patterns are deeply ingrained in an individual’s habitual way of interacting with the world.

## CLINICAL IMPLICATIONS

The group intervention, combining VR, BA, and CBT, is a novel and promising approach for patients with long-lasting dizziness. It offers a holistic treatment that addresses reaction patterns deeply embodied and cognitively learned to relate them to emotional aspects of dizziness. Participants gained insights into how fear, anxiety, and misinterpretations in exacerbating symptoms and learned strategies to confront previously avoided activities. This insight enhanced emotional resilience and better coping strategies even if participants acknowledged complete recovery as unattainable. The incorporation of peer support and shared learning seem to further enhance the intervention’s effectiveness and give a sense of community and validation of own experiences. These findings have the potential to inform the development of future interventions with long-lasting dizziness and contribute to improved patient outcomes, and the approach holds promise for integration in primary care clinical settings. The Figure gives a visual presentation of the impacts of the findings in our study. Physical therapists who work with VR have already reported incorporating anxiety management into their practice^48^ aligning with the bodily and cognitively based approach emphasized in our study. However, access to specialized psychological support for handling anxiety within VR is limited, as is VR in general. Therefore, addressing this gap in the integration of VR- and CBT-based interventions becomes imperative. Further research is required to determine the optimal number of treatment sessions and to further identify individuals who would benefit from this comprehensive treatment.

**Figure:**
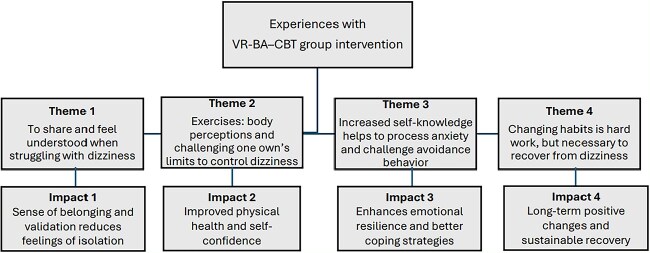
Clinical Impacts of the Core Findings After Participation in the Combined Program of Vestibular Rehabilitation, Body Awareness, and Cognitive Behavioral Therapy (VR-BA-CBT).

## Data Availability

Raw data were generated at The Western Norway University of Applied Sciences. Derived data supporting the findings of this study are available from the corresponding author on request.
